# Dietary FODMAP Manipulation for Exercise-Associated Gastrointestinal Symptoms: Why Symptom Severity Should Guide Our Approach

**DOI:** 10.1007/s40279-026-02474-9

**Published:** 2026-07-24

**Authors:** Rachel Scrivin, Natália Vilela Silva Daniel, Gary Slater, Ricardo J. S. Costa

**Affiliations:** 1 Faculty of Sport and Health, Toi Ohomai Institute of Technology, Tauranga, New Zealand; 2 School of Health, University of the Sunshine Coast, Sippy Downs, QLD Australia; 3 Faculdade de Ciências Aplicadas, Universidade Estadual de Campinas, Limeira, SP Brazil; 4 Faculty of Medicine Nursing and Health Sciences, Department of Nutrition Dietetics and Food, Monash University, Melbourne, VIC Australia

## Abstract

Exercise-induced gastrointestinal syndrome (EIGS) is a well-defined term that describes disruptions to gastrointestinal integrity and function that occur during exercise. A commonly reported outcome of EIGS is exercise-associated gastrointestinal symptoms (Ex-GIS), including flatulence, lower abdominal bloating and pain, and urge to defecate, which can compromise comfort, training quality and race-day execution. Low-fermentable oligo-, di-, mono-saccharide and polyol (FODMAP) diets are effective at reducing gastrointestinal symptoms in individuals with irritable bowel syndrome and, owing to similarities in symptom presentation, have increasingly been used by athletes to better manage Ex-GIS. This Current Opinion article argues that FODMAP manipulation should be used strategically rather than as a routine dietary default. For athletes with severe Ex-GIS, a short-term low-FODMAP approach may help attenuate symptoms around key training sessions and/or competition. In contrast, athletes with mild or infrequent symptoms may benefit from a more liberal FODMAP intake that mitigates disruptions to gastrointestinal integrity and potentially EIGS outcomes while avoiding unnecessary dietary restriction. However, neither approach has consistently been shown to improve performance. Accordingly, any modification of FODMAP intake should be individualized and guided by likely underlying primary mechanisms, symptom severity, exercise demands, and the need to maintain overall dietary adequacy.

## Key Points

**Table:** 

A low-FODMAP diet appears effective in reducing Ex-GIS severity, particularly when used short-term around key training sessions and/or competition.
Higher FODMAP intakes may mitigate EIGS pathophysiology with possible protective dietary influences; however, this may lead to greater nutrient malabsorption and Ex-GIS severity.
Current evidence does not show consistent performance benefits from short-term FODMAP manipulation.
Future research should incorporate standardized, validated symptomology assessment tools and full dietary provision in line with current best practice guidelines.

## Introduction

Exercise-associated gastrointestinal symptoms (Ex-GIS) are commonly reported by athletes, with up to 80% of ultra-endurance runners reporting symptoms, including nausea, urge to regurgitate, belching, upper-abdominal pain, urge to defecate, and diarrhea [1]. Ex-GIS can interrupt or prevent training (18.6%) and/or competitions (11.6%) [2] or, in some cases, be a sign of something more serious and of clinical concern aligned with exercise-induced gastrointestinal syndrome (EIGS), e.g., gastroparesis, acute reversible colitis, and/or septic shock [1, 3–6]. Ex-GIS are an outcome of EIGS [1, 7], which warrants attention with respect to both prevention and management strategies [8].

EIGS describes the complex pathophysiological response of the gastrointestinal tract to exercise that disrupts gastrointestinal integrity and/or function. EIGS develops via two primary causal pathways, the circulatory– and/or neuroendocrine–gastrointestinal pathways [8]. Two additional proposed pathways requiring further investigation include mechanical strain of exercise (i.e., jolting, jarring, and/or tissue friction) on the peritoneal cavity and exaggerated metabolic responses to exercise (i.e., decreased pH, or hypoglycemia) [8]. The minimum threshold of exertional stress required to promote substantial acute gastrointestinal integrity changes has been observed in experimental exercise models that apply ≥ 2-h exertional-heat stress (EHS) or ≥ 3-h in temperate conditions [9, 10]. Exercise stress below the minimum threshold is unlikely to lead to any clinically relevant changes to gastrointestinal integrity or function [10]. Specific therapeutic interventions with mostly consistent beneficial outcomes on Ex-GIS and gastrointestinal functional issues include promoting euhydration (e.g., before and during exercise), gut training, individual feeding tolerance adjustments, or the implementation of a low- fermentable oligo-, di-, mono-saccharide and polyol (FODMAP) diet [8].

FODMAPs are short-chain fermentable carbohydrates that are not entirely digested and/or poorly absorbed by the intestines [11, 12], whereby, upon reaching the large intestine, they are rapidly fermented by the colonic microbiota [11, 13]. Bacterial fermentation increases colonic contents, including water and gases such as hydrogen (H_2_), methane (CH_4_), and carbon dioxide (CO_2_) [14, 15]. The end point of fermentation may lead to an increase in short-chain fatty acids (SCFA) production, e.g., acetate, butyrate, and propionate [16]. Lowering dietary FODMAP content has proven efficacy for reducing gastrointestinal symptoms and improving quality of life for bowel disorders of gut–brain interaction, such as irritable bowel syndrome (IBS) [17–19]. Many of the gastrointestinal symptoms reported by people with IBS are similar to those described by athletes during and/or shortly after exercise, i.e., Ex-GIS [7]. It is not surprising that many athletes self-experiment with dietary management strategies to mitigate Ex-GIS, such as dietary fiber reduction (15%), dairy avoidance (6%), and low-FODMAP diets (5%), commonly implemented with success [20]. The most frequently eliminated FODMAP food components include lactose (88%), fructose (24%), galacto-oligosaccharides (22%), polyols (6%) and fructo-oligosaccharides (5%), with nearly all athletes reporting an improvement in their symptoms after specific dietary FODMAP removal [21].

However, as dietary FODMAPs have protective mechanisms for maintaining epithelial integrity (i.e., acting as a prebiotic) [16, 22] it is possible that a high-FODMAP diet has merit for endurance athletes in protecting the integrity of the gastrointestinal tract in response to prolonged strenuous exercise [22, 23]. Therefore, in this Current Opinion article, we argue that dietary FODMAP manipulation is a clinically useful intervention but one that requires careful consideration. Specifically, we propose that low-FODMAP diets are most appropriate for athletes experiencing frequent, performance-limiting symptoms, whereas higher-FODMAP diets can be used to support epithelial integrity and SCFA production when symptoms are less severe. We use available experimental data to support this position and recommend practical guidance on when to apply a low-FODMAP diet with endurance athletes.

## Study Selection

To inform this opinion, PubMed, SPORTDiscus, Ovid MEDLINE, CINAHL Ultimate, Embase, Cochrane reviews, and Web of Science databases were searched (July 2024) using terms related to endurance exercise, gastrointestinal symptoms, EIGS outcomes, and FODMAPs. We focused on experimental studies that applied a low-FODMAP diet before (e.g., 24–48-h) and/or during endurance exercise with either a high-FODMAP diet or a control, placebo, habitual or usual diet as a comparator. Studies excluded were animal models or in-vitro studies, and those that included athletes < 18 or > 55 years, pregnant and/or lactating females, sedentary individuals, or individuals with diagnosed gastrointestinal diseases or disorders (e.g., IBS and inflammatory bowel disease). Table 1 provides an overview of six experimental studies that implemented a low-FODMAP dietary intervention with endurance athletes to reduce EIGS and Ex-GIS. These studies applied an endurance exercise model, included a comparator to a low-FODMAP diet (e.g., high-FODMAP or habitual diet), and had a measurable outcome of EIGS and/or Ex-GIS. We used these studies to illustrate how varied FODMAP loads may influence EIGS outcomes and/or Ex-GIS, and how this should inform practical recommendations aligned with current best-practice guidelines [8].

**Table 1:** General characteristics of included studies investigating low-FODMAPs reducing EIGS and Ex-GIS (mean ± SD)

References	Participants	Study design	Intervention duration	Exercise protocol	Groups (FODMAP versus comparator)
Lis et al. [26]	M (*n* = 1) Recreationally competitive, multisport athlete History of Ex-GIS Age = 31 yr Hgt = 183.7 cm Wgt = 86.0 kg $\dot{V}$ O_2max_ = 57.9 mL·kg^−1^·min^−1^	Case study	14 days 6 days with a 1-day washout period in between subsequent 6 days	Same training (self-directed days 1, 2, 3, 7 and run days 4, 5, 6) Day 1, swim 60 min Day 2, cycle 60 min Day 3, rest Day 4, interval run 70 min Day 5, cycle 180 min and steady state run 65 min Day 6, interval run 65 min Day 7, not specified	6-day habitual versus 6-day low-FODMAP 1-day washout period in between Food plan provided Dietary compliance not confirmed Full FODMAP profile calculated
Lis et al. [29]	M (*n* = 5) F (*n* = 6) Recreationally competitive runners History of Ex-GIS Age = 41 ± 10 yr Hgt = 171.1 ± 10.0 cm Wgt = 69.0 ± 12.0 kg $\dot{V}$ O_2max_ = Ø	Randomized, single-blinded, cross-over	14 days 6 days with a 1-day washout period in between subsequent 6 days	Self-selected training schedule with days 4 and 5 prescribed Day 1 and 2, light to moderate intensity training Day 3, rest or very light non-running exercise Day 4, 5 × 1000 m run (100% predicted $\dot{V}$ O_2max_) Day 5, 7 km threshold run (90% predicted $\dot{V}$ O_2max_) Day 6, self-selected or rest Day 7, not specified	6-day low-FODMAP versus 6-day high-FODMAP 1-day washout period in between All food provided, with additional food exchanges Dietary compliance checked via food diaries Full FODMAP profile calculated
Wiffin et al. [32]	M (*n* = 6) F (*n* = 10) Recreationally active runners Age = 44 ± 10 yr Hgt = 170.0 ± 8.0 cm Wgt = 69.2 ± 8.8 kg $\dot{V}$O_2max_ = Ø	Randomized, single-blinded, cross-over	21 days 7 days with a 7-day washout period in between subsequent 7-day trial	Not specified, presumed self-selected running, 3 days a week	7-day low-FODMAP versus 7-day high-FODMAP 1-week washout in between Food lists provided Note: suggested food list provided with A (low-FODMAP) and B (high-FODMAP) food lists. Diet not controlled Dietary compliance checked via food diaries No FODMAP profile calculated
Gaskell et al. [23]	M (*n* = 10) F (*n* = 8) Recreationally competitive runners Age = 35 ± 8 yr Hgt = 174.0 ± 8.0 cm Wgt = 68.0 ± 12.0 kg $\dot{V}$O_2max_ = 62.0 ± 9.0 mL·kg^−1^·min^−1^	Randomized, controlled, double-blind, cross-over	12 days 1 × $\dot{V}$O_2max_ testing day, and 2 trials each with a 24 h lead-in and trial day. 7-day washout between trials	2-h running at 60% $\dot{V}$ O_2max_ (HFOD = 35.8 °C ± 1.0 °C, 22.6 ± 1.8% RH and LFOD = 35.7 °C ± 0.9 °C, 23.3 ± 3.2% RH)	24-h low-FODMAP versus 24-h high-FODMAP 1-week washout in between All food provided, homogeneous packaging, similar foods/drinks provided Compliance checked via dietary logs Full FODMAP profile calculated
Scrivin et al. [28]	M (*n* = 10) F (*n* = 2) Recreationally competitive endurance athletes Age = 42.0 ± 5.2 yr Hgt = 174.0 ± 8.0 cm Wgt = 72.1 ± 13.7 kg $\dot{V}$O_2max_ = 53.2 ± 8.6 mL·kg^−1^·min^−1^	Randomised, controlled, double-blind, cross-over	14 days 1× $\dot{V}$O_2max_ testing day, and 2 trials each with a 48-h lead-in and trial day. 7-day washout between trials	2-h running at 60% of $\dot{V}$ O_2max_ in temperate ambient conditions (22.9 ± 1.2 °C, 46.4 ± 7.9% RH), followed by a 1-h distance performance test	48-h high carbohydrate low-FODMAP versus 48-h high carbohydrate high-FODMAP 1-week washout in between All food provided, homogeneous packaging, very similar foods/drinks provided Dietary compliance checked via food diaries Full FODMAP profile calculated
Scrivin et al. [31]	Athletes with Ex-GIS: M (*n* = 5)/F (*n* = 4) Controls, without Ex-GIS: M (*n* = 1)/F (*n* = 1) Recreationally competitive to elite runners Age = 41 ± 11 yr Hgt = 176.0 ± 10.0 cm Wgt = 75.6 ± 17.0 kg $\dot{V}$O_2max_ = Ø	Case series	2–3 months (1 × assessment day with several months follow up)	3-h treadmill running at self-selected race pace in temperate ambient conditions (19.8 ± 1.1 °C, 64 ± 7% RH)	Habitual versus 48-h high carbohydrate low-FODMAP Food plan provided Dietary compliance checked via food diaries Full FODMAP profile calculated

cm, centimeter; EIGS, exercise-induced gastrointestinal syndrome; Ex-GIS, exercise-induced gastrointestinal symptoms; F, female; FODMAP, fermentable oligo-, di-, mono-saccharide and polyol; g∙d^−1^; grams per day; h, hour; hgt, height; kg, kilogram; m, meter; M, male; MSUM, multistage ultra-marathon; ml·kg·min^−1^, milliliters per kilogram per minute; *n*, number; RDBCT, randomized double-blind control trial, RH, relative humidity; SD, standard deviation; yr, year; $\dot{V}$O_2max_; maximal oxygen update; wgt, weight; °C, degrees Celsius; %, percentage; Ø, no results or specific data

## Dietary FODMAP Manipulation

The recommended low-FODMAP dietary cutoffs have been defined as < 0.5 g FODMAPs at each sitting (e.g., meal and/or snack) [24, 25], and the efficacy of these cutoffs has been tested in several studies [11, 24]; however, study participants were either individuals with IBS or healthy controls, not specifically endurance athletes. As endurance athletes have increased energy and carbohydrate requirements, it is likely that this leads to a higher FODMAP intake, e.g., up to 81 g per day (g·d^−1^) of total FODMAPs reported in an endurance athlete’s habitual diet [26, 27], compared with ~ 24 g·d^−1^ in a typical Australian diet [24]. It is reasonable to assume that owing to an endurance athlete’s increased energy and carbohydrate requirement, a more realistic low-FODMAP diet cutoff is < 5 g·d^−1^ total FODMAPs, which is recommended for exercise gastroenterology research [9].

When comparing the low-FODMAP dietary interventions, of the six included studies, only three studies provided adequate dietary control where all intervention food and drink were provided to promote dietary adherence, with dietary intake confirmed against food diaries (Table 2) [23, 28, 29]. The dietary controlled low- and high-FODMAP trials comprised 2.0 ± 0.7 g·d^−1^ versus 46.9 ± 26.2 g·d^−1^ [23], 3.0 ± 0.2 g·d^−1^ versus 54.8 ± 10.5 g·d^−1^ [28], 8.1 ± 3.5 g·d^−1^ versus 41.4 ± 7.9 g·d^−1^ [29] of total FODMAPs, respectively. Although the low-FODMAP trial in Lis et al. [29] exceeded the commonly recommended cutoff of < 5 g·d^−1^ total FODMAPs, the absolute difference between low- and high-FODMAP diets remained large enough to constitute clear dietary exposures, justifying their comparison within this context. [9]. Taken together, we believe the data support the pragmatic use of a short-term low-FODMAP intervention for athletes reporting substantial Ex-GIS, particularly when full dietary provision and adherence can be achieved. However, considering only two studies implemented robust dietary control aligned to best-practice guidelines (e.g., a randomized double-blind crossover protocol with full dietary provisions), this highlights that our current position rests on limited evidence, and that clinical decisions must therefore be individualized rather than protocol-driven. In advocating for low-FODMAP strategies, we acknowledge the potential for restrictive dietary practices to exacerbate disordered eating in susceptible athletes [30]. We view low-FODMAP manipulation as a time-limited, symptom-driven intervention that should be used with expert nutrition support and careful monitoring, allowing for adequate energy availability.

**Table 2:** Dietary components of included studies investigating low-FODMAPs reducing EIGS and Ex-GIS (mean ± SD)

References	Groups	Total energy (kcal∙d^−1^)	Total CHO (g∙d^−1^ or g∙kg·d^−1^)	Total protein (g∙d^−1^)	Total fat (g∙d^−1^)	Total fiber (g∙d^−1^)	Total FODMAPs (g∙d^−1^)
Lis et al. [26]	Low FODMAP	2527 ± 407	303 ± 69 (~ *3.5*)	122 ± 24	85 ± 28	33 ± 6	7.2 ± 5.7^a^
	Habitual	2586 ± 416	295 ± 68 (~ *3.4*)	136 ± 9	88 ± 22	34 ± 9	81.0 ± 5.0
Lis et al. [29]	Low FODMAP	3198 ± 429	327 ± 47 or (~ *4.7*)	153 ± 20^a^	137 ± 15^a^	30 ± 5	8.1 ± 3.5^a^
	High FODMAP	3181 ± 403	323 ± 63 or (~ *4.7*)	158 ± 16	130 ± 12	32 ± 5	41.4 ± 7.9
Wiffin et al. [32]	Habitual	2356 ± 197	246 ± 19 or (*3.6* ± *0.2*)	92 ± 8	102 ± 13	Ø	28.0 ± 4.3
	Low FODMAP	1999 ± 138	194 ± 22 or (*2.8* ± *0.3*)^a^	94 ± 6	89 ± 10	Ø	15.8 ± 1.9^a,b^
	High FODMAP	2269 ± 162	272 ± 26 or (*3.9* ± *0.4*)	95 ± 7	84 ± 6	Ø	38.6 ± 6.5
Gaskell et al. [23]	Low FODMAP	2375 ± 538^a^	355 ± 89 or (*5.2* ± *1.0*)^a^	91 ± 23^a^	66 ± 15	50 ± 9	2.0 ± 0.7^a^
	High FODMAP	2645 ± 747	405 ± 124 or (*6.0* ± *1.5*)	102 ± 29	68 ± 18	53 ± 16	46.9 ± 26.2
Scrivin et al. [28]	Habitual	3000 ± 595	329 ± 93 (*4.6* ± *1.2*)	140 ± 36	114 ± 28	34 ± 15	21.7 ± 15.8
	High carbohydrate. low FODMAP	3746 ± 659^a,b^	657 ± 128 or (*9.1* ± *1.0*)^a,b^	121 ± 22^a^	63 ± 11^a^	42 ± 3^b^	3.0 ± 0.2^a,b^
	High carbohydrate, high FODMAP	4006 ± 498^b^	704 ± 98 or (*9.8* ± *1.3*)^b^	141 ± 21	62 ± 8^a^	41 ± 5^b^	54.8 ± 10.5^b^
Scrivin et al. [31]	High carbohydrate, low FODMAP	3459 ± 582	592 ± 140^a^	106 ± 14^a^	67 ± 13^a^	30 ± 5	4.5 ± 3.2^a^
	Habitual	2953 ± 1472	295 ± 211	177 ± 87	112 ± 58	36 ± 12	28.6 ± 17.0

EIGS, exercise-induced gastrointestinal syndrome; Ex-GIS, exercise-induced gastrointestinal symptoms; g∙d^−1^, grams per day; g∙kg·d^−1^, grams per kilogram per day; FODMAP, fermentable oligo-, di-, mono-saccharide and polyol; kcal∙d^−1^, kilocalorie per day; SD, standard deviation; ± , plus or minus; Ø, no results or specific data

^a^Significant difference between low-FODMAP and high-FODMAP (*p* < 0.05)

^b^Significant difference to habitual diet (*p* < 0.05)

## Effect of Dietary FODMAP Manipulation on Ex-GIS

To reduce the risk of bias when assessing Ex-GIS, the use of a validated visual analogue scale during exercise is advocated [7], alongside pretest familiarization [7, 9]. Despite this a range of symptom assessment tools have been used to measure and describe Ex-GIS. Three studies [23, 28, 31] used a validated visual analogue scale [7] recommended for exercise gastroenterology research [9], whereas the other studies used nonstandardized and nonvalidated scales [26, 29, 32]. Across studies, lower Ex-GIS severity during exercise was reported in most low-FODMAP trials (*n* = 5), compared with high-FODMAP trials (Table 3). Specifically, low-FODMAP trials were associated with less gut discomfort [23, 28], total GIS severity [23, 28, 32], upper GIS severity [23, 28], lower GIS severity [23], nausea, and dizziness [23]. Collectively, these findings support our positioning that low-FODMAP diets reduce Ex-GIS severity. In contrast, Lis et al. [29] reported no differences in Ex-GIS severity between trials; however, the exercise stress load was likely insufficient to result in substantial Ex-GIS, as demonstrated in other exercise models that reliably promoted Ex-GIS [33–36]. From a practical perspective, we interpret these findings as evidence that low-FODMAP diets reduce Ex-GIS severity rather than the incidence per se. That is, athletes may still experience Ex-GIS during exercise, but when they do, the symptom severity appears to be reduced when low-FODMAP strategies are implemented.

**Table 3:** Effect of varied FODMAP diets on the incidence (during exercise and recovery) and severity (during exercise) of exercise-associated gastrointestinal symptoms

References	Assessment tool	Trials	Incidence during exercise	Incidence in recovery	Total gut discomfort	Total GIS	Total upper GIS	Total lower GIS	Nausea	Dizziness	Stitch
Lis et al. [26]	Questionnaire 0–9 au	Low FODMAP	Ø^a^	Ø^a^	0	0	0	0	0	Ø	0
		Habitual	Ø^a^	Ø^a^	20 au (2–4 au)	20 au (0–4 au)	4 au (0–2 au)	13 au (0–4 au)	0	Ø	3 au (0–3 au)
Lis et al. [29]	Questionnaire 0–9 au	Low FODMAP	Ø	Ø	Ø	Ø	Ø	Ø	Ø	Ø	Ø
		High FODMAP	Ø	Ø	Ø	Ø	Ø	Ø	Ø	Ø	Ø
Wiffin et al. [32]	VAS 0–100 au Endpoint data	Low FODMAP	Ø^a^	Ø^a^	Ø	31.3 ± 9.2^b^ au	Ø	Ø	Ø	Ø	Ø
		High FODMAP	Ø^a^	Ø^a^	Ø	104.0 ± 25 au	Ø	Ø	Ø	Ø	Ø
Gaskell et al. [23]	mVAS 0–10 au Mean (summative range)	Low FODMAP	88%	59%	9^b^ (2–31)	17^b^ (2–98)	8^b^ (1–56)	8^b^ (1–42)	1^b^ (3–12)	1^b^ (8)	0 (1–2)
		High FODMAP	88%	82%	17 (1–34)	49 (1–247)	21 (1–110)	24 (4–128)	3 (1–26)	2 (1–16)	1 (4–8)
Scrivin et al. [28]	mVAS 0–10 au Summative (individual range)	HC low FODMAP	92%	58%	119^b^ (2–25)	220 (3–75)	88^b^ (3–15)	75 (0–40)	12 (2–8)	9 (1–6)	13 (2–11)
		HC high FODMAP	100%	58%	190 (2–36)	314 (2–75)	136 (1–24)	139 (0–41)	12 (6)	4 (4)	15 (2–7)
Scrivin et al. [31]	mVAS 0–10 au	HC low FODMAP	Ø^a^	Ø^a^	Ø^a^	Ø^a^	Ø^a^	Ø^a^	Ø^a^	Ø^a^	Ø^a^
		Habitual	100%	100%	269 (10–59)	480 (14–117)	90 (0–29)	213 (0–98)	46 (0–16)	35 (0–17)	8 (0–8)

Au, arbitrary unit; GIS, gastrointestinal symptoms; g∙d^−1^, grams per day; g∙kg·d^−1^, grams per kilogram per day; FODMAP, fermentable oligo-, di-, mono-saccharide and polyol; HC, high carbohydrate; kcal∙d^−1^, kilocalorie per day; mVAS, modified visual analog scale; ± , plus or minus; Ø, no results or specific data

^a^Self-reported improved symptoms

^b^Significant difference between low-FODMAP and high-FODMAP (*p* < 0.05)

## Effect of Dietary FODMAP Manipulation on Gastrointestinal Integrity

The circulatory–gastrointestinal pathway of EIGS describes a redistribution of blood flow away from the splanchnic area toward the exercising muscles and peripheral circulation, to aid skeletal muscle metabolism and thermoregulation during exercise [1, 8]. Reduced blood flow to the splanchnic area leads to perturbations of the intestinal epithelium, contributing to gastrointestinal dysfunction and symptom development [1, 8]. A key biomarker used to measure the magnitude of intestinal epithelial damage is plasma intestinal fatty acid-binding protein (I-FABP) concentrations [9]. I-FABP is solely present in the enterocytes of the intestinal epithelium and is a proposed surrogate marker indicative of enterocyte damage [37].

Only two studies that manipulated dietary FODMAP load have measured changes in plasma I-FABP concentrations at rest and in response to exercise (Table 4) [23, 28]. For clinical relevance, the minimal detectable change (MDC) of pre- to post-exercise plasma I-FABP concentrations is ≥ 1301 pg∙mL^−1^ [10]. Scrivin et al. [28] observed no significant differences in pre- to post-exercise plasma I-FABP concentration between dietary interventions (i.e., high-carbohydrate low-FODMAP ~ 601 pg·mL^−1^; high-carbohydrate high-FODMAP ~ 706 pg·mL^−1^). In this instance, it is likely that the 48-h high carbohydrate intake (i.e., 10–12 g·kg·d^−1^) pre-exercise, and modest carbohydrate (30 g·h^−1^) during the first 2 h of steady-state exercise, lessened epithelial injury, regardless of dietary FODMAP intake [28]. Similarly, higher carbohydrate intakes (i.e., 45 g·h^−1^) during exercise can ameliorate epithelial injury [38] and attenuate small intestine permeability [39]. These findings support the importance of carbohydrate ingestion to maintain carbohydrate availability to fuel exercise while also mitigating intestinal epithelial damage [38, 40, 41]. In contrast, Gaskell et al. [23] had participants running for 2 h at 60% of $\dot{V}$ O_2max_ in 35 °C heat without carbohydrate intake during exercise, which resulted in clinically relevant pre-to post-exercise changes in plasma I-FABP concentrations of ~ 1805 pg·mL^−1^ and ~ 1174 pg·mL^−1^ on low- and high-FODMAP, respectively. It is likely that lower plasma I-FABP concentrations observed on high-FODMAP are due to greater nutrient content along the intestinal lumen, as evidenced by higher breath H_2_ during the recovery period and/or greater fecal SCFA concentrations, which may stimulate epithelial microvascular blood flow, thereby mitigating intestinal epithelial injury [22, 23].

**Table 4:** Effect of varied FODMAP diets on gastrointestinal integrity and functional responses to endurance exercise

References	Participants	Exercise protocol and dietary protocol	Gastrointestinal integrity I-FABP (pg·mL^−1^) Mean ± SD or mean (95% CI)	Gastrointestinal function Malabsorption (ppm) OCTT (min) Mean (95% CI)
Wiffin et al. [32]	M (*n* = 6) F (*n* = 10) Recreationally active runners	Exercise protocol: Not specified, presumed self-selected running, 3 days a week Dietary protocol: 7-day low-FODMAP versus 7-day high-FODMAP 1-week washout in between	Pre and post-intervention: Low-FODMAP pre: 206.9 ± 7.3 and post: 219.5 ± 10.4 High-FODMAP pre: 218.2 ± 10.9 and post: 222.6 ± 13.1 ↑6% pre- to post-exercise low-FODMAP ↑2% pre- to post-exercise high-FODMAP ↔ Pre- to post-intervention low-FODMAP ↔ Pre- to post-intervention high-FODMAP ↔ Between trials	Ø
Gaskell et al. [23]	M (*n* = 10) F (*n* = 8) Recreationally competitive runners	Exercise protocol: 2-h running at 60% $\dot{\mathrm{V}}$ O_2max_ in 35 °C ± , 23% RH Dietary protocol: 24-h low-FODMAP versus 24-h high-FODMAP 1-week washout in between All food provided	Pre- and post-exercise: Low-FODMAP pre: 263 (162–364) and post: 2068 (1078–3058) High-FODMAP pre: 255 (176–334) and post: 1429 (872–1987) ↑710% pre- to post-exercise low-FODMAP ↑443% pre- to post-exercise high-FODMAP Main effect of trial ↑ I-FABP levels high-FODMAP (*p* = 0.033) ↑ magnitude of ∆ pre- to post-high-FODMAP (*p* = 0.088)	Malabsorption of CHO ↑H_2_ in recovery for high-FODMAP ↑AUC for high-FODMAP = 2525 (1452–3597) versus low-FODMAP (1031–1978)
Scrivin et al. [28]	M (*n* = 10) F (*n* = 2) Recreationally competitive	Exercise protocol: 2-h running at 60% of $\dot{\mathrm{V}}$ O_2max_ in temperate ambient conditions (22.9 ± 1.2 °C, 46.4 ± 7.9% RH), followed by a 1-h distance performance test Dietary protocol: 48-h high-carbohydrate low-FODMAP versus 48-h high-carbohydrate high-FODMAP 1-week washout in between All food provided	*Pre- to post-exercise:* High carbohydrate low-FODMAP pre: 519 (371–674) and post: 1120 (749–1508) High carbohydrate high-FODMAP pre: 442 (233–660) and post: 1148 (709–1607) ↑116% pre- to post-exercise high-carbohydrate low-FODMAP ↑160% pre- to post-exercise high-carbohydrate high-FODMAP ↔ Between trials ↔ No main effect of trial (*p* = 0.675) ↔ No difference in magnitude of ∆ pre- to post-exercise (*p* = 0.781)	OCTT (min) High carbohydrate low-FODMAP = 95 (64–126) High carbohydrate high-FODMAP = 83 (50–115) Note: no turning point after 180 min High carbohydrate low-FODMAP = 1 participant High carbohydrate high-FODMAP = 2 participants

AUC, area under the curve; CHO, carbohydrate; CI, confidence interval; EIGS, exercise-induced gastrointestinal syndrome; Ex-GIS, exercise-induced gastrointestinal symptoms; F, female; g∙d^−1^, grams per day; g∙kg·d^−1^, grams per kilogram per day; FODMAP, fermentable oligo-, di-, mono-saccharide and polyol; H_2_, hydrogen; h, hour; I-FABP, intestinal fatty acid binding protein; kcal∙d^−1^, kilocalorie per day; M, male; mins, minutes; *n*, number; OCTT, orocecal transit time; pg·mL^−1^, picograms per milliliter; ppm, parts per million; RH, relative humidity; SD, standard deviation; $\dot{V}$O_2max,_ maximal oxygen update; °C, degrees Celsius; %, percentage; ± , plus or minus; ↔ , no difference; ↑, increase; ∆, change; Ø, no results or specific data

Supporting this rationale, secondary sample analysis by Gaskell et al. [22] demonstrated that a high-FODMAP diet (51 g·d^−1^) compared with a low-FODMAP diet (2 g·d^−1^) substantially increased total fecal SCFA (i.e., + 60%), including acetate, butyrate, and propionate concentrations, as well as modestly increasing plasma total SCFA (i.e., + 16%) and acetate concentrations. Scrivin et al. [42] reported similar findings, i.e., high-carbohydrate high-FODMAP diets were associated with significantly higher total fecal SCFA (i.e., + 48%), including acetate and butyrate concentrations compared with habitual diets. These findings support the rationale for SCFA contributing to reduced intestinal epithelial injury, despite a higher fermentative load [22].

Owing to perturbations in intestinal epithelial integrity experienced during exercise, it is likely that bacteria translocate into the systemic circulation, causing systemic inflammatory and/or anti-inflammatory cytokine responses [9]. However, only two studies investigated secondary follow-on systemic outcomes [23, 28]. Both Gaskell et al. [23] and Scrivin et al. [28] found no significant differences in systemic responses between low- and high-FODMAP trials (Table 5). It is important to note that laboratory models using exercise stress with or without carbohydrate intake may not be sufficient to elicit substantial systemic cytokine responses. Moderate increases in cytokine responses have only been observed in three laboratory studies [33, 35, 43], whereas field studies consistently report much larger increases [44, 45]. It is possible that no effect was observed in Gaskell et al. [23] and Scrivin et al. [28] as much greater exercise stress is required to truly detect intervention effects at the systemic cytokine level. However, as dietary FODMAPs maintain epithelial integrity (i.e., acting as a prebiotic and subsequent SCFA production) [16, 22], it is possible that a high-FODMAP diet has merit for endurance athletes in protecting the integrity of the gastrointestinal tract in response to prolonged strenuous exercise [22, 23]. We argue that low-FODMAP diets are best positioned as short-term strategies to reduce Ex-GIS severity. Once acute symptom control is achieved, practitioners should prioritize the reintroduction of FODMAP-containing foods where tolerated, particularly for endurance athletes with high training loads, to support gastrointestinal integrity and ensure overall dietary adequacy.

**Table 5:** Effect of varied FODMAP diets on systemic immunological responses to endurance exercise

Reference	Participants	Exercise protocol and dietary protocol Mean ± SD	Systemic responses Mean (95% CI)
Gaskell et al. [23]	M (*n* = 10) F (*n* = 8) Recreationally competitive runners	Exercise protocol: 2-h running at 60% $\dot{\mathrm{V}}$ O_2max_ in heat (35 °C ± , 23% RH) Dietary protocol: 24-h low-FODMAP versus high-FODMAP diet 1-week washout in between All food provided	LBP and sCD14 (μg∙mL^−1^) Ø ↔ No main effect of trials ↔ magnitude of ∆ pre- to post-exercise between diets (*p* = 0.088) ↔ magnitude of ∆ pre- to post-exercise between diets sCD14 ↔ (*p* = 0.478) Cytokines (pg·mL^−1^) Ø *↑* modest IL-10 main effect over time on both high-FODMAP and low-FODMAP ↔ No main effect of trials for IL-1β, TNF-α, IL-6, IL-8 ↔ No magnitude of ∆ pre- to post-exercise for IL-1β, TNF-α, IL-6, IL-8, IL-10
Scrivin et al. [28]	M (*n* = 10) F (*n* = 2) Recreationally competitive	*Exercise protocol:* 2-h running at 60% of $\dot{V}$O_2max_ in temperate ambient conditions (22.9 ± 1.2 °C, 46.4 ± 7.9% RH), followed by a 1-h distance performance test *Dietary protocol:* 48-h high-carbohydrate low-FODMAP diet versus 48-h high-carbohydrate high-FODMAP 1-week washout in between All food provided	sCD14 (ng·mL^−1^) HC-low-FODMAP pre-exercise = 2396 (2064–2742) HC-low-FODMAP 1-h post-exercise peak = 2992 (2685–3313) HC-high-FODMAP pre-exercise = 2562 (2085–3060) HC-high-FODMAP 2-h post-exercise peak = 3200 (2627–3800) ↔ No main effect of trials (*p* = 0.897) ↔ No magnitude of ∆ pre- to post-exercise between diets (*p* = 0.664) CRP (μg·mL^−1^) High-carbohydrate low-FODMAP pre-exercise = 5.1 (3.3–7.1) High-carbohydrate low-FODMAP 2-h post-exercise peak = 6.0 (4.0–8.1) High-carbohydrate high-FODMAP pre-exercise = 5.1 (3.0–7.4) High-carbohydrate high-FODMAP 2-h post-exercise peak = 6.0 (3.5–8.6) ↔ No main effect of trials (*p* = 0.832) ↔ No magnitude of ∆ pre- to post-exercise between diets (*p* = 0.368)

CRP, C-reactive protein; CI, confidence interval; F, female; FODMAP, fermentable oligo-, di-, mono-saccharide and polyol; h, hour; IL, interleukin; LBP, lipopolysaccharide binding protein; M, male; ng.mL^−1^, nanograms per milliliter; n, number; *μ*g·mL^−1^, micrograms per milliliter;* p*, probability; pg·mL^−1^, picograms per milliliter; sCD14, soluble cluster differentiation-14; SD, standard deviation; RH, relative humidity; TNF, tumor necrosis factor; $\dot{V}$O2_max,_ maximal oxygen update; °C, degrees Celsius; %, percentage; ± , plus or minus; ↔ , no difference; ↑, increase; ∆, change; α, alpha; β, beta; Ø, no results or specific data

## Effect of Dietary FODMAP Manipulation on Gastrointestinal Function

The development of EIGS via the neuroendocrine gastrointestinal pathway is due to an increase in sympathetic nervous system activity, which affects gastrointestinal functional capacity (i.e., impaired gastrointestinal motility and malabsorption) [1]. Gastrointestinal function can be assessed using various techniques to establish gastrointestinal emptying, transit, absorption, and smooth muscle activity [9]. Less-invasive techniques include a lactulose challenge, ingestion of a telemetry pill, measuring breath H_2_ and CH_4_, or electrogastrography [9]. To date, only two studies have investigated gastrointestinal function in the context of FODMAP manipulation during exercise, assessing either carbohydrate malabsorption [23] or orocecal transit time (OCTT) [28].

Carbohydrate malabsorption, typically determined by an increase in breath H_2_ responses > 10 ppm from baseline [46], is clinically relevant to Ex-GIS given its association with luminal gas production and an increase in osmotic fluid, which are potential triggers of Ex-GIS during exercise. Gaskell et al. [23] reported significantly greater area under the curve for breath H_2_ concentration during exercise and recovery following a high-FODMAP diet compared with a low-FODMAP diet (*p* = 0.031) (Table 4) [23]. This is unsurprising given FODMAPs are osmotically active and readily fermented, naturally producing more H_2_ [11, 24]. A high-FODMAP diet is likely to increase luminal contents, which may have further contributed to carbohydrate malabsorption [13]. Malabsorbed carbohydrate might activate the ileal brake, delaying gastrointestinal transit, and increasing nutrient exposure within the intestine during exercise, potentially mitigating intestinal hypoperfusion [47]. In contrast, Scrivin et al. [28] reported delayed OCTT in both high-FODMAP and low-FODMAP trials (~ 83 min and ~ 95 min post exercise, respectively), which was an unexpected finding given the lower fermentable loads in the low-FODMAP trial. As both trials included a 48-h high-carbohydrate loading period, it is plausible that increased total energy and food volume increased luminal contents, thereby influencing gastrointestinal motility equally, rather than the FODMAP load per se. This observation could have broader implications for endurance athletes adhering to carbohydrate-loading strategies independently of FODMAP intake. Given this limited and somewhat equivocal data on gastrointestinal transit and carbohydrate malabsorption, we do not believe current evidence supports using FODMAP manipulation primarily to ‘optimize’ gut function. However, reductions in carbohydrate malabsorption and fermentation provide a biologically plausible pathway by which low-FODMAP diets reduce Ex-GIS symptom burden during exercise. These mechanistic effects likely operate through reductions in luminal distention and gas production, rather than improvements in motility as such. We suggest that FODMAP manipulation should be viewed as a symptom targeted strategy that may benefit susceptible athletes, rather than a universal prescription, reinforcing the need for individualized case-by-case application.

## Effect of Dietary FODMAP Manipulation on Performance Outcomes

While it might be assumed that any moderation of Ex-GIS severity and/or incidence would have the potential to result in favorable performance outcomes, only one experimental study [28] has explored the performance implications of low-FODMAP diets. Endurance athletes consumed a 48-h high-carbohydrate high-FODMAP or high-carbohydrate low-FODMAP diet with a standardized low-FODMAP breakfast before completing a 2-h steady-state run at 60% of $\dot{V}$ O_2max_ followed by a 1-h distance performance test in temperate ambient conditions. Despite greater Ex-GIS severity reported on the high-carbohydrate high-FODMAP trial, no significant difference was observed for the distance completed during the 1-h distance performance test [28]. In contrast, field research in events > 3-h has indicated self-reported performance improvements on a 48-h high-carbohydrate low-FODMAP diet [31]. In real-world settings, a higher carbohydrate diet before prolonged endurance events is advocated to optimize glycogen storage, which can enhance athletic performance and delay fatigue [48] Given that only one controlled trial and a small number of case-series indicate possible performance benefits when low-FODMAP strategies are used as part of a broader gastrointestinal management strategy, we do not consider it appropriate to advocate low-FODMAP diets as a performance-enhancing intervention in their own right. Rather, our position is that any performance gains are likely to be secondary to better symptom control in selected athletes and should be communicated as such to avoid unrealistic expectations.

## Conclusions

In this Current Opinion article piece, we have argued that dietary FODMAP manipulation is a useful but nuanced tool for managing Ex-GIS in endurance athletes. Only a small number of well-controlled experimental trials suggest that 24–48-h low-FODMAP diets can reduce the severity of Ex-GIS during and after exercise, whereas high-FODMAP diets may better support epithelial integrity, likely via their prebiotic effects. We view this apparent paradox not as a contradiction but as an indication that FODMAP manipulation should be used strategically, rather than simplistically.

For athletes with frequent, severe, performance-limiting Ex-GIS, we advocate a short-term, practitioner-led low-FODMAP diet around key training sessions and/or events, accompanied by specific carbohydrate provision during exercise to mitigate epithelial injury. For athletes with mild or infrequent symptoms, we see greater merit in a high-FODMAP diet, which may have some protective effect on intestinal epithelial integrity, aligned with its prebiotic properties (i.e., increased fecal and/or SCFA concentrations) [22]. In all cases, we emphasize that low-FODMAP diets should not become a chronic default diet in endurance sport, especially for those with restrictive eating patterns or disordered eating [30]. Dietary FODMAP manipulation should be viewed as one component of a broader, individualized gastrointestinal management plan. This plan should be preceded by an appropriate assessment to identify likely causal pathways and implemented in collaboration with qualified practitioners. Future research using best practice methodological frameworks is required to test and refine this opinion, but we support the principles outlined, which provide a pragmatic basis for clinical decision-making.

## Declarations

### Availability of data and material

Data are available from the corresponding author if requested.
